# The expanding landscape of regulated cell death

**DOI:** 10.1016/j.mmr.2026.100060

**Published:** 2026-08-07

**Authors:** Dao-Lin Tang, Rui Kang

**Affiliations:** Department of Surgery, UT Southwestern Medical Center, Dallas, TX 75390, USA

**Keywords:** Biology, Regulated cell death (RCD), Damage-associated molecular patterns, Disease, Plasticity

The field of regulated cell death (RCD) is entering a new phase. Over the past decade, the RCD landscape has expanded far beyond apoptosis, first described in 1972, to encompass a growing repertoire of mechanistically distinct modalities, including necroptosis, pyroptosis, ferroptosis, and several newly emerging forms of cell death [1], [2], [3] (Fig. 1). Yet the significance of these discoveries extends well beyond the addition of new terminology. Collectively, they have transformed cell death from a collection of molecular pathways into a unifying principle of physiology and disease. Rather than representing the passive endpoint of cellular injury, RCD is now recognized as an active process that orchestrates tissue homeostasis, immune regulation, development, and pathological progression. In this sense, the field has evolved from identifying individual death pathways to redefining the biological roles of cell death itself.

**Fig. 1:**
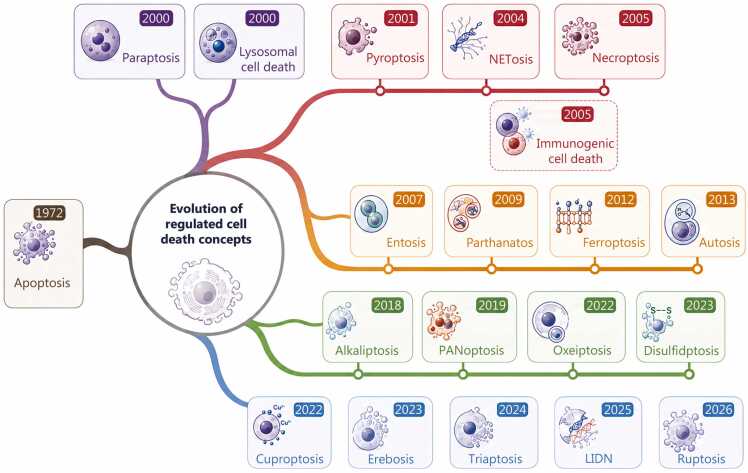
**Evolution of regulated cell death concepts.** Timeline illustrating the emergence of major regulated cell death modalities from apoptosis (1972) to recently proposed forms of cell death. Years indicate when each concept was first introduced. LIDN. Lipid-dependent necrosis.

Perhaps the most important conceptual advance is the realization that individual death pathways rarely function in isolation. Classifying RCD into distinct modalities has provided an essential framework for elucidating their molecular mechanisms [4]. However, simply expanding the catalog of death modalities is unlikely to drive the next breakthrough. In many physiological and pathological settings, multiple death programs coexist, share upstream stress signals, converge on common metabolic and inflammatory checkpoints, and compensate for one another under selective pressure. Concepts such as PANoptosis further illustrate that pyroptosis, apoptosis, and necroptosis can operate as coordinated rather than independent programs [5]. The challenge now is no longer to define additional forms of cell death, but to uncover the principles that integrate diverse death pathways into coherent cellular responses. Among these emerging principles, metabolic dysregulation is beginning to emerge as a common denominator across seemingly distinct forms of RCD, raising the possibility that disruption of metabolic homeostasis, rather than individual signaling pathways alone, represents a fundamental determinant of cellular fate [6].

This shift in perspective has profound implications for disease biology. The biological significance of cell death is determined not simply by whether cells die, but also by the signals generated during the dying process. Different forms of RCD are therefore defined not only by their molecular execution but also by the distinct immunological and metabolic landscapes they create. Through the release of damage-associated molecular patterns (DAMPs), cytokines, metabolites, and other immunomodulatory signals, dying cells actively shape immune activation, tissue remodeling, and intercellular communication. Consequently, the immunogenicity of cell death has emerged as a key determinant of therapeutic outcome, particularly in cancer, where DAMP-driven activation of innate and adaptive immunity contribute to the efficacy of immunotherapy [7]. Conversely, uncontrolled activation of inflammatory or oxidative death programs contributes directly to tissue injury in neurodegenerative disorders, ischemia-reperfusion injury, and chronic inflammatory diseases [8]. Emerging evidence further suggests that these signals can propagate stress responses and secondary cell death to neighboring cells, indicating that RCD should be viewed not merely as a cell-autonomous process but as a coordinated event operating at the tissue level [9]. Understanding the context in which a particular death program is engaged may therefore prove as important as deciphering the molecular machinery that executes it.

This conceptual framework also calls for a reassessment of therapeutic strategies. Considerable effort has focused on selectively activating or inhibiting individual death pathways, yielding encouraging preclinical and early clinical results. Nevertheless, the remarkable plasticity of cell death programs suggests that targeting a single pathway is often insufficient. Cells frequently evade one death mechanism by engaging alternative survival or death programs, limiting the durability of therapeutic responses. Future interventions will likely depend less on manipulating individual pathways than on rationally coordinating interconnected cell death programs according to disease context, with the ultimate goal of restoring tissue homeostasis rather than simply inducing or preventing cell death. This transition from pathway-specific intervention to network-based modulation may ultimately define the next generation of cell death-directed therapies.

Several fundamental questions now define the frontier of the field. Do newly identified forms of RCD represent truly distinct biological entities, or are some better understood as context-dependent manifestations of shared cellular stress responses? How should overlapping molecular signatures be interpreted in heterogeneous tissues? More importantly, how can mechanistically relevant cell death be distinguished from secondary pathological consequences *in vivo*? Addressing these questions will require integrating mechanistic biology with quantitative approaches capable of resolving cell death in its native spatial and temporal context. Equally important will be the development of reliable biomarkers that faithfully reflect specific death programs in patients and enable precision therapeutic intervention.

The next decade of RCD research is unlikely to be defined by the discovery of yet another cell death modality. Instead, the major advances will come from understanding how different death programs cooperate, compete, and converge on common metabolic and immunological principles that shape disease evolution and therapeutic response. Advances in single-cell and spatial technologies are now making it possible to reconstruct dynamic cell death networks within intact tissues, while studies of the interplay between RCD, immunity, metabolism, aging, and tissue regeneration continue to uncover new therapeutic opportunities.

Ultimately, the expanding landscape of RCD reflects more than an increase in nomenclature, it represents a fundamental shift in biological thinking. The future of the field will not be measured by the number of newly defined death modalities, but by our ability to integrate mechanistic insights into a coherent understanding of cellular fate and translate that knowledge into clinically meaningful strategies for precision intervention [10].

## Abbreviations

RCDRegulated cell death

DAMPsDamage-associated molecular patterns

## Ethics approval and consent to participate

Not applicable.

## Funding

This work was supported by the US National Institutes of Health (GM127791 to DLT and DK144077 to RK). DLT is also supported by the American Cancer Society (MBGI-25-1423399-01-MBG).

## Data Availability

Not applicable.
